# Penicillin allergy delabeling in a German University Hospital—implementation in inpatient settings: the PenGuin Pilot Study

**DOI:** 10.1007/s15010-026-02915-7

**Published:** 2026-08-11

**Authors:** Hannah Nürnberg, Katrin Hörth, Kristin Egenberger, Stefanie Amelung, Elena Jaszkowski, Julia Bauer, Carolin Deiß, Ute Chiriac, Alexandra Weber, Anne Wiesner, Britta Kölking, David Martin, Miriam Pasinato, Tilman Schöning, Claudia M. Denkinger, Benedict Morath, Elham Khatamzas

**Affiliations:** 1 Hospital Pharmacy, University Hospital Heidelberg, Heidelberg, Germany; 2 Department of Infectious Diseases and Tropical Medicine, University Hospital Heidelberg, Heidelberg, Germany; 3 German Center of Infection Research, Partner site Heidelberg, Braunschweig, Germany

**Keywords:** Penicillin allergy, Delabeling, Implementation, Antibiotic stewardship

## Abstract

**Background:**

Penicillin allergy (PenA) is reported in up to 15% of hospitalized patients, although true IgE-mediated hypersensitivity is rare. PenA delabeling is an important antimicrobial stewardship intervention, as mislabeling is associated with adverse clinical outcomes. While structured delabeling interventions are safe and effective, their translation into routine care across different healthcare settings remains unclear.

**Methods:**

This prospective implementation study was conducted on four wards of a 2600-bed German tertiary care center. All adult inpatients with reported PenA underwent structured allergy assessment and risk stratification by clinical pharmacists. Low risk patients were delabeled directly, while moderate-risk patients were evaluated for direct oral challenge (DOC). Outcomes included delabeling rate at discharge, ward-specific differences, and implementation barriers and facilitators assessed by staff questionnaires. Sustainability was evaluated by primary care follow-up 32 weeks after discharge.

**Results:**

Among 2890 screened patients, 179 (6.2%) reported a PenA. Of 162 eligible patients, 54 (33.3%) were successfully delabeled. DOC was well tolerated, with only one mild skin reaction observed. Ward-specific environments influenced the use of different delabeling strategies. Key barriers included staffing shortages, short hospital stays, and patient-related factors, whereas facilitators included easy integration into pharmacist workflows and interprofessional collaboration. Follow-up showed limited transfer of delabeling outcomes to primary care, reducing long-term sustainability.

**Conclusion:**

Pharmacist-led PenA delabeling can be safely integrated and is feasible within existing clinical workflows. However, real-world implementation barriers and incomplete transfer to primary care limited its full potential. Improve standardized documentation and cross-sector communication are important for sustainable and widespread implementation of delabeling strategies.

**Supplementary Information:**

The online version contains supplementary material available at https://doi.org/10.1007/s15010-026-02915-7.

## Introduction

Penicillin allergy (PenA) labels affect up to 15% of hospitalized patients, although most of these labels are not confirmed as true immune-mediated allergies [1]. Mislabeling has significant consequences for patient care, including increased use of second-line antibiotics, higher rates of antimicrobial resistance, longer hospital stays, as well as higher treatment costs [2–6]. Consequently, penicillin allergy delabeling (i.e. removing false penicillin allergy labels) has become part of antimicrobial stewardship programs worldwide [7]. International data, primarily from the United States, the United Kingdom, and Australia, have shown that structured delabeling interventions are both safe and effective, even when performed by non-allergists such as pharmacists and ward physicians [8–12]. Within interprofessional antimicrobial stewardship programs, pharmacists are well positioned to initiate and coordinate structured penicillin allergy assessments and delabeling [13]. Studies on delabeling demonstrate that standardized risk stratification with tools like the PEN-FAST score, combined with direct oral provocation testing for selected patients, can successfully remove incorrect penicillin allergy labels [14–17]. However, despite growing evidence, knowledge gaps remain such as the sustained translation and implementation of penicillin allergy delabeling strategies into different healthcare systems. A recent systematic review demonstrated variability in delabeling strategies and effectiveness across settings, suggesting that successful delabeling depends on local structures and available resources [18]. Within the German healthcare setting it is however unclear how such interventions can be feasibly integrated into clinical inpatient routine without additional staffing or structural resources. Prospective implementation studies are missing in non-prescribing pharmacists setting, and documentation practices, clinical responsibilities, and availability of allergy services vary across institutions [19]. In addition, the long-term sustainability of delabeling, particularly for the time after discharge into primary care remains unexplored for Germany. Thus, this study aimed to address these knowledge gaps by providing the first real-world data on the implementation of a pharmacist-led structured delabeling intervention in a German university hospital. This study focuses on impact of interprofessional collaboration, intersectoral communication, as well as barriers and facilitators of implementation.

## Methods

### Setting

This prospective quality improvement study was conducted at a German tertiary care center with 2.600 beds. The study involved four wards where clinical pharmacists are members of the interprofessional team. Each ward is covered by one clinical pharmacist on weekdays (Monday to Friday) who performs medication reconciliation for all elective patients and participates in daily interdisciplinary ward rounds or case discussions together with physicians and nursing staff. Participating wards included cardiac and vascular surgery, ear-nose-throat (ENT), and radio-oncology.

### Study design

This prospective implementation study was conducted in accordance with the Standards for Quality Improvement Reporting Excellence (SQUIRE) guideline between July 8th and November 30th, 2024 [20]. Patient recruitment and data collection were conducted during this period. The study was designed to provide a basis for future multicenter implementation. Adult patients (18 years and older) with a reported penicillin allergy were eligible for inclusion and were identified during routine care on participating wards. The delabeling intervention was integrated into existing clinical workflows without additional personnel or financial resources. Participation of patients and healthcare professionals was voluntary. In the case of healthcare professionals, participation was voluntary in regard to implementation evaluation, including completing questionnaires and interviews. As the implementation of the delabeling intervention followed a local institutional guideline, physicians were able to perform delabeling procedures regardless of whether they participated in the evaluation of implementation.

### Intervention description

**Fig. 1:**
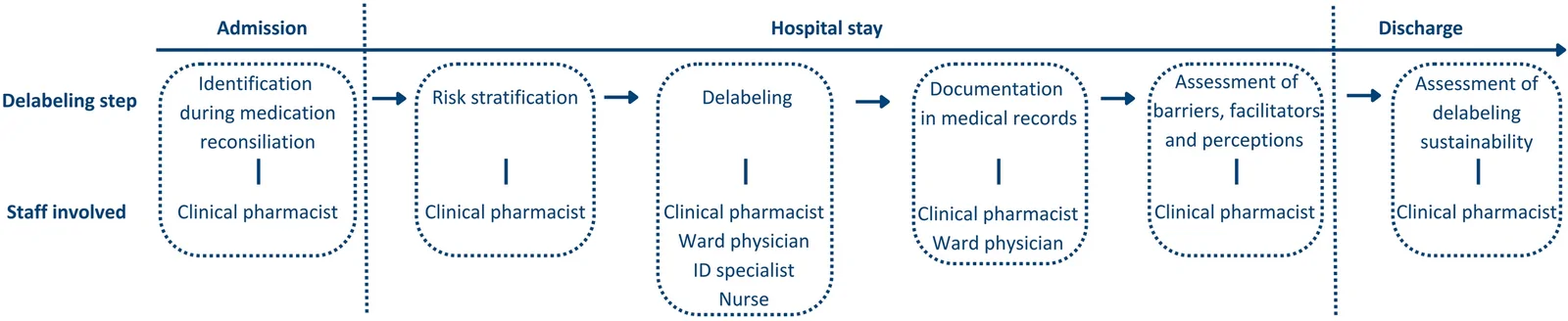
Penicillin allergy delabeling process. *ID: *Infectious diseases

The penicillin allergy delabeling process is shown in Fig. 1. Clinical pharmacists identified eligible patients during daily routine (medication reconciliation, ward rounds) and performed allergy history assessment and risk stratification using the local guideline of the antimicrobial stewardship team (Supplementary 1). Based on this assessment, clinical pharmacists carried out direct delabeling without further allergy testing for patients classified as low risk. Patients eligible for direct delabeling were not routinely reviewed by an infectious disease specialist. All patients classified as moderate risk were referred to infectious disease specialists for evaluation and final decision to perform a direct oral challenge (DOC). Eligible patients were prescribed a single dose of Amoxicillin 500 mg orally by ward physicians after informed consent following standardized protocols (supplementary 2). Prior to study initiation, ward physicians and nursing staff on participating wards were introduced to the study protocol. Education was based on the institutional PenA delabeling guideline. They were educated on the rationale and potential benefits of delabeling and were advised to monitor for signs and symptoms of allergic reactions for one hour after DOC. Physicians were educated during weekly infectious diseases ward rounds by the responsible clinical pharmacist and infectious disease specialist. Nursing staff was educated as part of their regular ward-based continuing education sessions by the responsible clinical pharmacist. Delabeling outcomes were documented in medical records by clinical pharmacists. Discharge documentation had to be updated by ward physicians using documentation aids provided by the local guideline.

### Outcomes and data collection

The primary outcome of this study was the proportion of patients who were successfully delabeled either by direct delabeling or successful DOC resulting in a removed PenA label at discharge following the intervention. Secondary clinical outcomes included the characterization of reported index reactions and the categorization of patients into risk groups according to the internal guideline (supplementary 1). Data were collected using the structured questionnaire of the internal guideline, which were completed paper-based by clinical pharmacists during routine care (supplementary 3). Implementation outcomes focused on barriers and facilitators influencing the PenA delabeling process. These were assessed by structured questionnaires distributed to healthcare professionals (i.e. clinical pharmacists, ward physicians, infectious disease specialists, nurses) during the implementation period and were categorized according to the Consolidated Framework for Implementation Research (CFIR) [21] (supplementary 4). The questionnaire included open-ended questions on workload, feasibility, and factors facilitating or constraining implementation in routine clinical practice. Responses were mapped to the corresponding CFIR domains to provide a structured framework for reporting the implementation findings. Sustainability was evaluated by assessing whether the PenA label remained removed in primary care documentation systems. For this purpose, primary care physicians were contacted after patient consent via telephone and fax and were asked to completed a standardized follow-up questionnaire (supplementary 5). All data were pseudonymized and stored securely in institutional databases in accordance with data protection regulations.

### Statistical analysis

Descriptive statistics were used to summarize patient characteristics and implementation outcomes. Categorical variables were presented as counts and percentages, continuous variables using median and interquartile range (IQR). Analyses were performed using Microsoft Excel 2021 (version 2108). Differences in prevalence and delabeling outcomes between participating wards were evaluated using exploratory Chi-square tests. Statistical significance was defined as *p* < 0.05. Sample size for prevalence analysis was calculated to estimate the prevalence of reported PenA with adequate precision. Assuming a prevalence of 7.5%, a 95% confidence level (Z = 1.96), and a desired absolute precision (half-width of the CI) of 0.02, a minimum of 666 screened patients was required. As a quality improvement study, the sample size for the primary delabeling outcome was pragmatic and based on the number of eligible inpatients during the study period on participating wards. The delabeling rate is reported with 95% confidence intervals using the exact Clopper-Pearson method.

### Ethics approval and consent to participate

The ethics committee of Heidelberg University Hospital approved the study (positive vote S-512/2023). All procedures were in accordance with the declaration of Helsinki.

## Results

### Admission and prevalence

During the study period, 179 of 2890 screened patients, admitted to the participating wards, reported a PenA during medication reconciliation, resulting in an overall prevalence of 6.2% for the included wards. The highest prevalence was detected in cardiac surgery (35/391, 9.0%), followed by radio-oncology (37/474, 7.8%). ENT wards and vascular surgery showed lower prevalence of reported penicillin allergy (ENT: 90/1564, 5.8%; vascular surgery: 17/461, 3.7%) (*p* = 0.005).

### Reported reactions

**Table 1:** Reported reaction types

Reaction types	Number of patients (*n* = 179)
Non-immune mediated reactions	*n* = 24 (13.4%)
Isolated nausea/ vomiting/ diarrhoea	*n* = 12
Others	*n* = 7
In combination with other symptoms	*n* = 5
Unknown/ not memorable	*n* = 24 (13.4%)
Rash and Urticaria	*n* = 108 (60.3%)
Isolated diffuse rash	*n* = 79
Delayed rash	*n* = 9
Rash with blistering	*n* = 7
In *combination* with other symptoms	*n* = 13
Respiratory swelling/ bronchospasm	*n* = 16 (8.9%)
Isolated respiratory symptoms	*n* = 8
In *combination* with other symptoms	*n* = 8
Hypotension	*n* = 10 (5.6%)
Isolated Hypotension	*n* = 3
Unexplained collapse	*n* = 3
In *combination* with other symptoms	*n* = 4
Diffuse swelling	*n* = 5 (2.8%)
Prick-test positive	*n* = 2 (1.1%)
Angioedema	*n* = 1 (0.6%)
Anaphylactic shock	*n* = 1 (0.6%)
Combinations	
Rash + gastrointestinal symptoms	*n* = 2
Rash + bronchospasm	*n* = 3
Rash + bronchospasm + hypotension	*n* = 1
Rash + diffuse swelling	*n* = 7
Bronchospasm + hypotension	*n* = 2
Bronchospasm + vomiting	*n* = 1
Bronchospasm + vomiting + Fever	*n* = 1
Hypotension + diarrhea + vomiting	*n* = 1

Reaction types assessed during allergy assessment are shown in Table 1. The most reported reactions to penicillin antibiotics were skin rash (108/179; 60.3%), followed by non-immune mediated reactions like isolated nausea (24/179; 13.4%) and respiratory symptoms (16/179, 8.9%). A minor proportion of patients reported diffuse body swelling (5/179, 2.8%), prior positive prick-test (2/179, 1.1%), angioedema (1/179, 0.6%) and anaphylactic shock (1/179, 0.6%). Twenty-four patients (13.4%) were unable to characterize the nature of their reaction. Combinations of reaction types were reported in 18 patients (10.0%).

### Risk stratification

**Fig. 2:**
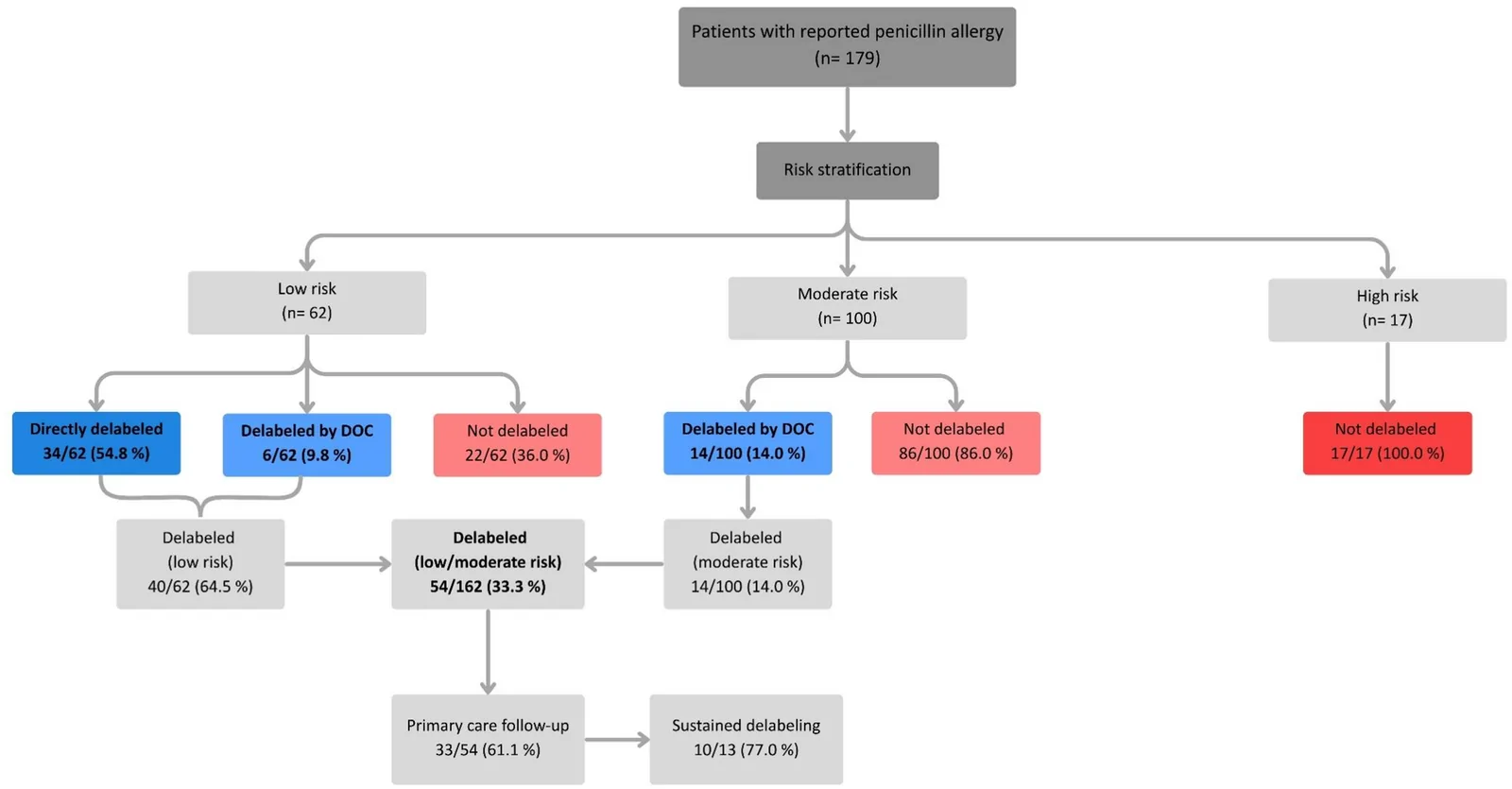
Risk stratification according to local guideline and delabeling outcomes. *DOC:* direct oral challenge; Directly delabeled: Delabeled by allergy history without testing; Sustained delabeling: Patients without penicillin allergy label in primary care follow up according to 13 returned questionnaires

Patients were risk stratified according to the local penicillin allergy management guideline. Sixty-two patients (34.6%) were classified as low risk, 100 patients (55.9%) as moderate risk, and 17 patients (9.5%) as high risk of true penicillin allergy (Fig. 2). Time since index reaction was more than five years ago in 138 patients (77.1%), 38 (21.2%) of the reactions occurred during childhood. Overall five patients (2.8%) could not remember the time of the allergy reaction. The PEN-FAST score supported the clinical risk stratification, details can be found in the supplemental material 6.

### Delabeling outcomes

Overall, 54 of 162 eligible patients (33.3%, 95% CI 26.1–41.2%) were delabeled following the delabeling intervention (Fig. 2). Based on allergy history, 34 of 62 (54.8%) low risk patients were directly delabeled without further testing. The remaining 21 patients underwent DOC. Of these, six patients were classified as low risk and 15 as moderate risk according to the local guideline. DOC was well tolerated in all but one patient, who developed an immediate, non-severe rash not requiring treatment. This patient had been classified as moderate risk by the local guideline and following the reaction retained the allergy label. Delabeling outcomes were documented in discharge letters for 47 of 54 delabeled patients (87.0%).

### Ward differences

**Fig. 3:**
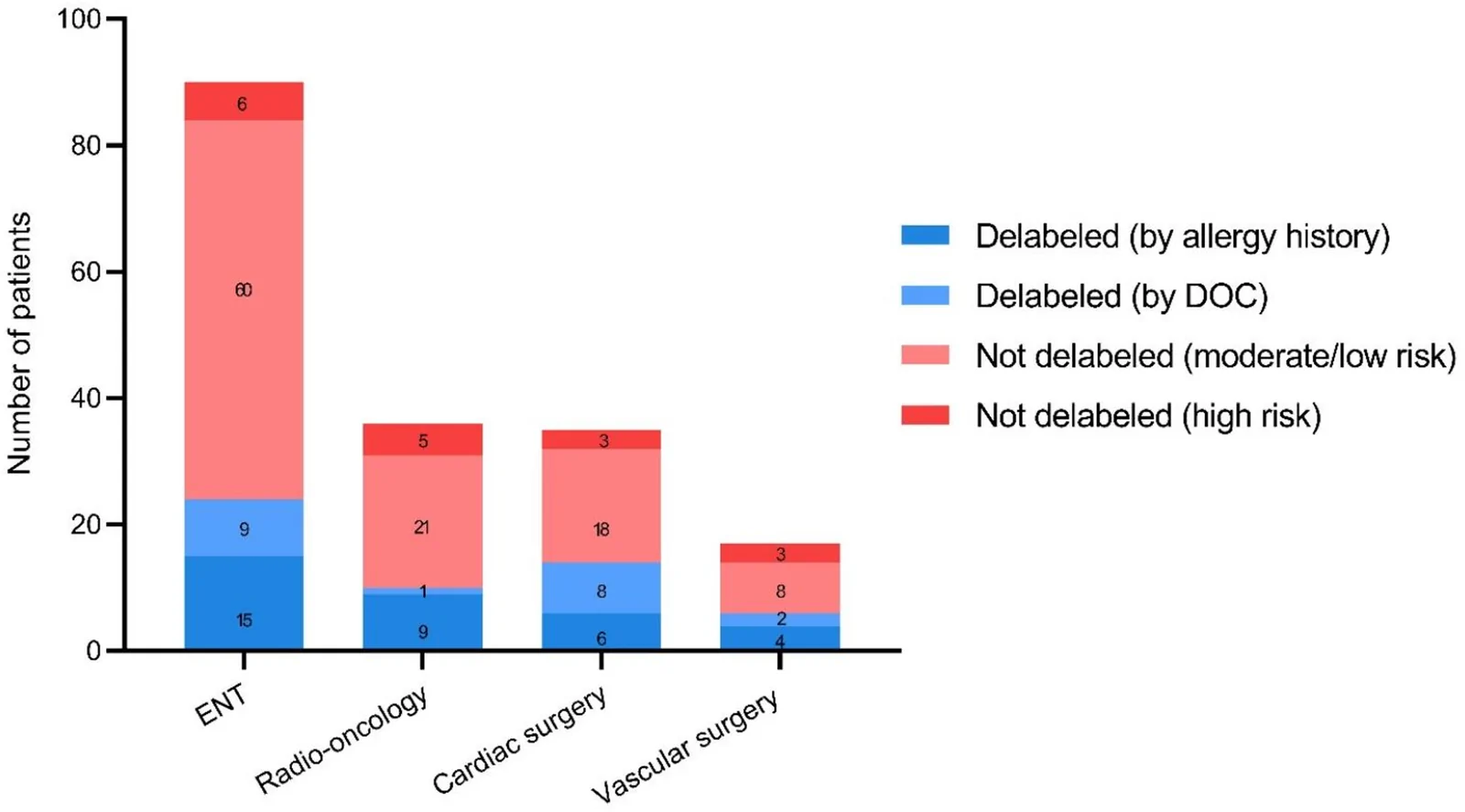
Use of delabeling strategies stratified by ward. *ENT:* ear nose throat

Stratification by ward demonstrated differences in the relative use of delabeling strategies (Fig. 3). On ENT and radio-oncology wards, a larger proportion of patients were directly delabeled based on allergy history alone (ENT: 15/24, 62.5%; radio-oncology: 9/10, 90.0%), while in cardiac surgery a higher proportion of patients underwent direct oral challenge (8/14, 57.1%). In vascular surgery, both approaches were used less frequently, reflecting the smaller overall number of eligible patients. Delabeling rate was highest in cardiac surgery (14/35, 40.0%) and lowest in ENT (24/90, 26.7%). Differences in delabeling rates across participating wards were not significant (*p* = 0.473).

### Barriers and facilitators

Implementation of PenA delabeling was influenced by a combination of facilitating and limiting factors. All identified barriers, including reasons for unsuccessful delabeling, as well as facilitators are summarized and mapped to the CFIR framework in Table 2.

Fifteen healthcare professionals completed the questionnaire on barriers and facilitating factors. The most frequently reported facilitators included interdisciplinary collaboration (10/15, 66.7%), the beneficial role of pharmacists in direct delabeling and coordinating the process (6/15, 40.0%), and training for ward staff (4/15, 26.7%). The availability of a local guideline with well-structured workflows (3/15, 20.0%), and easy integration of delabeling into routine medication assessment conducted by clinical pharmacists (3/15, 20.0%) further facilitated the delabeling process. Recurring barriers identified by healthcare professionals were related to limited medical staffing resources (6/15, 40.0%), high workload (6/15, 40.0%), short length of hospital stays (8/15, 53.3%), and patient-related factors such as language barriers, uncertainty about undergoing PenA delabeling, or refusal of delabeling (6/15, 40.0%). Structural and process-related challenges (6/15, 40.0%), particularly during the early implementation phase, further affected feasibility.

Overall, 125 of 179 patients (69.8%) could not be delabeled. Seventeen of them (13.6%) were classified as high risk according to the internal guideline and therefore not eligible for delabeling. Reasons for unsuccessful delabeling varied between wards. On the ENT ward, delabeling most often failed due to short hospital stays with discharge before completion of evaluation, combined with a high patient turnover and overall high workload. In radio-oncology, delabeling was frequently not pursued for clinical reasons (palliative situation, systemic corticosteroid therapy, critical clinical condition). In addition, ward physicians were initially partly sceptical towards delabeling, citing inexperience with assessing and managing allergic reactions. Acceptance of delabeling increased among ward physicians after targeted education. Across all wards, however, the most frequent reasons preventing delabeling were high-risk classification and staffing shortages among the involved pharmacists and infectious disease specialists, which limited the ability to provide patient education and perform testing.

**Table 2:** Barriers and facilitators mapped to the Consolidated Framework for implementation research (CFIR)

CFIR Domain	CFIR Construct	Facilitators	Barriers
Intervention characteristics			
	*Relative Advantage*	Direct delabeling based on allergy history easier than oral challenge	
	*Design Quality & Packaging*	Standardized questionnaire for allergy assessment Easy identification of eligible patients during medication reconciliation	
	*Complexity*	Easy integration of allergy assessment into medication reconciliation Uncomplicated consultation of infectious disease specialists (email/ telephone)	Infectious disease specialist not continuously present on the ward (consult required) Longer duration of medication assessment
Outer Setting			
	*Patient Needs & Resources*	Patients are grateful for evaluation and time Patients understand purpose after education Positive perception of delabeling process in general	Language barriers Patient refusal (fear of allergic reaction, lack of understanding delabeling purpose, patient’s conviction of having a true allergy, uncertainty about allergy history) Critical medical conditions Palliative care setting (radio-oncology)
	*External Policies & Incentives*		Pharmacists are not allowed to prescribe and perform oral provocation testing
Inner Setting			
	*Structural Characteristics*		Admission/Discharge during weekends Staffing shortages (pharmacists, infectious disease specialists) High additional workload
	*Networks & Communication*		Lack of clear referral guidance from pharmacists to infectious disease specialists during the early implementation phase, resulting in over-referral and information overload
	*Implementation Climate*	Low turnover settings with longer hospital stays (e.g. Cardiac surgery) – more time for delabeling	High turnover settings with short hospital stay (ENT)
	*Readiness for Implementation*		Lack of initial education on penicillin allergy delabeling purpose and management for ward physicians
Characteristics of individuals			
	*Knowledge & Beliefs*	Ward physicians trusted pharmacists’ allergy assessment and risk stratification	Refusal or skepticism among clinical staff to perform delabeling (lack of understanding relevance or importance)
	*Self-Efficacy*	Acceptance of delabeling increased among ward physicians after targeted education	
Process			
	*Planning*	No facilitators and barriers were identified from questionnaire responses	
	*Engaging*	Pharmacist-led coordination of the process Consulting of infectious disease specialists Education of ward physicians to support infectious disease specialists in times of staffing shortage	
	*Executing*		Discharge prior to evaluation or testing Postponed or cancelled surgical procedures

*CFIR:* Consolidated Framework for implementation research; *ENT: *ear nose throat

### Follow up

Out of 54 delabeled patients, 33 (61.1%) agreed to follow-up with their primary care physician after eight months. A total of 13 questionnaires (39.4%) from primary care physicians were returned to the study team. In 10 of 13 patients (76.9%), the penicillin allergy status was “no penicillin allergy” in the primary care documentation. In nine of these 10 cases (90.0%), the primary care physician previously did not know about the patient’s penicillin allergy. Three patients (23.1%) had already been prescribed a penicillin antibiotic after hospital discharge. One patient reported a mild allergic reaction characterized by unspecific body swelling. However, the patient stated that they would take penicillin in the future if medically indicated.

Nine of 13 (69.2%) primary care physicians stated that they would be willing to prescribe a penicillin antibiotic to the patient in the future. Regarding discharge documentation, 3 of 13 (23.1%) primary care physicians reported that the delabeling was documented in the hospital discharge letter, whereas five physicians (38.5%) reported not having received a discharge letter, and another five (38.5%) reported that the delabeling was not documented. On review, the absent of documentation regarding the delabeling intervention could be confirmed in only one hospital record, suggesting that four of the five physicians likely did not receive or read the discharge letter. One physician stated that the patient herself reported on the delabeling.

## Discussion

This pilot study represents the first real-world evaluation of a pharmacist-led penicillin allergy delabeling program implemented in inpatient clinical practice in Germany. The findings of this study demonstrate that penicillin allergy delabeling can be embedded into routine patient care. However, implementation under routine conditions was influenced by context-specific factors including personnel resources, duration of hospital stay, and patient-related aspects, which varied across participating wards. As a pilot study, it provides an important foundation for future multicenter studies and broader implementation within antimicrobial stewardship (AMS) programs.

The participating wards had previously been included in a separate point-prevalence analysis conducted at our institution. The prevalence observed during the implementation study (6.2%) was slightly lower than the previously detected overall prevalence of 8.1%. This difference is presumably explained by normal temporal variation. Overall, the prevalence aligns with international data (5–15%) and highlights the relevance of delabeling initiatives in the German healthcare setting [1, 22].

The overall delabeling rate observed in our study was 33%, with 63% of these patients being directly delabeled based on structured allergy history alone by pharmacists and 37% following direct oral challenge (DOC). When considering only patients eligible for history-based delabeling, more than half (55%) could be successfully delabeled. International pharmacist-led programs have reported wide ranges of delabeling rates (37–80%), reflecting differences in patient selection and clinical setting [23–25]. Nevertheless, a substantial proportion of patients in our study could not be delabeled despite low risk stratification (20/62, 32%). This is likely because our program was implemented entirely within clinical practice without any additional personnel and financial resources, dedicated research staff, and controlled conditions. The results may therefore better reflect realistic expectations for real-world implementation. Barriers identified in this study align with those reported in international studies, including high clinical workload, limited staffing and time resources, clinical concerns regarding safety of delabeling, and lack of confidence to perform PenA delabeling [26, 27]. Patient related barriers identified in this study included fear of an allergic reaction, uncertainty about allergy history or about undergoing delabeling, and critical clinical conditions. This aligns with patient related barriers in previous studies reporting critical illness, cognitive or emotional overload during hospitalization, and fear of an allergic reaction [18, 28]. In contrast, key facilitators for successful delabeling identified in this study included productive interprofessional collaboration, mutual trust between professional groups, clear task allocation, and targeted education for both staff and patients. Our findings are in line with previous international studies, which have highlighted the importance of involving clinical pharmacists, expanding non-allergist-led programs, and integrating structured education and training into established AMS workflows to facilitate PenA delabeling in clinical routine [26, 29, 30].

A key finding of our study was the substantial difference in employed delabeling strategies across participating wards. These differences may reflect ward-specific patient populations and workflows. While direct delabeling based on allergy history was more frequently used in radio-oncology and ENT wards, DOC was more commonly performed in primarily surgical wards. Although these factors were not formally assessed, the higher proportion of palliative patients typically encountered in radio-oncology and the shorter length of hospital stay typically observed in high turnover settings such as ENT may have limited the feasibility and acceptance of DOC. In contrast, longer hospital stays on surgical wards may have facilitated the planning and conduction of DOC. These findings suggest that penicillin allergy delabeling strategies may require adoption to local clinical environment, including patient populations and local workflows [18]. Differences in risk perception among healthcare professionals may also have influenced the observed variation of delabeling strategies between wards. However, as this aspect was not evaluated, this interpretation remains speculative. Direct delabeling was easier to implement than DOC and required less resources, suggesting that prioritizing direct delabeling in low-risk patients may be an feasible strategy for high workload settings.

Consistent with previous studies, a high proportion of patients reported unknown or poorly described index reactions [1, 31]. These findings support the growing evidence that a significant proportion of penicillin allergy labels can be safely removed without additional testing after appropriate risk stratification and therefore represented an ideal target population for pharmacist-led interventions [2, 8, 32].

In this setting, clinical pharmacists played a major role in the delabeling process supporting their potential role within delabeling programs embedded within AMS-teams. This is particularly relevant for the German healthcare system where pharmacists have no independent prescribing authority. While pharmacists can initiate and coordinate delabeling processes and perform direct delabeling in accordance with local guidelines, clinical decision making, prescribing of antibiotics for DOC and diagnostic procedures such as skin testing remain the responsibility of physicians and infectious disease or allergy specialists.

Although based on responses from a limited proportion of primary care physicians, the exploratory primary care follow-up revealed preliminary insights into challenges regarding the sustainability of PenA delabeling after hospital discharge. These challenges are consistent within previously published studies: While PenA labels are updated correctly in hospital records, documentation in primary care often remains inconsistent, with many patients still carrying their PenA label after being delabeled or avoiding penicillin antibiotics despite negative testing [33]. In this study, discharge letters were not received by some of the patients’ primary care physicians. In recent studies incomplete discharge communication and absence of shared documentation systems between hospital and primary care have also been identified as possible contributors [34]. These findings suggest that loss of information between healthcare sectors may compromise the long-term sustainability of inpatient delabeling interventions. Strategies such as standardized patient-held delabeling documentation and integration of delabeling into electronic patient records may help improve the visibility of delabeling outcomes beyond hospitalization. In this context, the recent nationwide rollout of the electronic patient record in Germany may offer an opportunity to improve cross-sector documentation in the future.

Our findings have several implications for the future implementation in Germany. First, standardized training programs, delabeling algorithms and documentation practices are needed. Further, penicillin allergy delabeling should be considered for integration into AMS programs, with clear definition of responsibilities, prioritization strategies, and a central role for pharmacists in allergy assessment, interprofessional coordination and targeted education. In settings with limited resources, prioritizing patients classified as low-risk and eligible for direct delabeling without further testing may be beneficial as this approach is less time and resource intensive. Alternatively, prioritizing patients who are currently receiving or require antibiotic therapy may maximize clinical benefit.

Future research should focus on multicenter studies to confirm generalizability, assess clinical outcomes, and evaluate long-term sustainability. Integration in electronic patient records and follow-up beyond hospital discharge are likely to support long-term sustainability.

## Limitations and strengths

This study has several limitations. First, the sample size of this study was limited, and the exploratory design did not include confirmatory analyses or formal sample size calculation. Additionally, ward-specific differences may limit generalizability. Second, this was a single-centre study conducted in a tertiary care university hospital with access to infectious disease specialists and clinical pharmacists. Therefore, transferability to smaller hospitals without on-site infectious disease specialists or clinical pharmacists may be limited. Implementation strategies may need to be adapted to local structures and available expertise. Third, parts of this intervention and follow-up required patient consent and willingness of primary care physicians to provide information, which may lead to selection and response bias. As a result, patients who are more open towards delabeling, as well as perspective of physicians interested in AMS and PenA delabeling may be overrepresented. In terms of evaluating implementation, the use of brief questionnaires rather than focus groups provided less detailed insights into implementation barriers and facilitators. These limitations as well as small sample size should be considered when interpreting the follow-up findings. Finally, this intervention was planned involving pharmacists and physicians and not solely infectious diseases specialists. The role of other professional groups like nursing staff, medical students or outpatient allergy services was not evaluated and may present an area for future research.

Despite these limitations, this study has several strengths. To our knowledge, this is the first study evaluating the implementation of pharmacist-led penicillin allergy delabeling in Germany. It provides relevant real-world insights into the implementation of pharmacist-led penicillin allergy delabeling in routine inpatient care. Furthermore, the follow-up after hospital discharge enabled assessment of the sustainability of the intervention and highlighted the importance of intersectoral communication. The multidisciplinary approach involving pharmacists and physicians and not solely infectious disease specialists also reflects practical clinical conditions and supports broader implementation in Germany.

## Conclusion

Structured allergy assessment enabled efficient identification and direct delabeling of low-risk patients within existing clinical workflows. However, delabeling rates and long-term sustainability were limited by real-world implementation barriers and challenges in communication beyond hospital discharge. Pharmacists can play a crucial role in coordinating delabeling and supporting interprofessional collaboration. To further support pharmacist-led interventions institutional support, standardized documentation, and integration into AMS programs will be important. Overall, this pilot real-world implementation study provides preliminary evidence supporting the feasibility of a pharmacist-led penicillin allergy delabeling in routine inpatient care in Germany. Future multicenter implementation studies are needed to evaluate the generalizability of these findings and to support the widespread implementation of standardized, scalable penicillin allergy delabeling strategies in German healthcare settings.

## Supplementary Information

Below is the link to the electronic supplementary material.

Supplementary Material 1

Supplementary Material 2

## Data Availability

The datasets used and analysed during the current study are available from the corresponding author on reasonable request.
