# Supplementary material for: Penicillin allergy delabeling in a German University Hospital—implementation in inpatient settings: the PenGuin Pilot Study
Source: Infection. 2026 Aug 11;54(5):2715–24. doi: 10.1007/s15010-026-02915-7 (PMC13727940; doi:10.1007/s15010-026-02915-7)
Supplement: Supplementary file 1 [file 15010_2026_2915_MOESM1_ESM.pdf]

SUPPLEMENTARY

Supplementary 1: Local guideline for penicillin allergy management available to hospital staff on local intranet

1. **Penicillin Allergy History Form**

▪ Which penicillin antibiotic was not tolerated (do you know the exact name)?

▪ What condition was present at the time of drug intake, or what was the indication for antibiotic therapy?

▪ When did the reaction occur? Within the last 5 years? More than 10 years ago?

▪ What was the nature of the reaction? Symptoms? Anaphylaxis?

- Was treatment required? If yes, please specify:
- In case of exanthema: mucosal involvement, blistering, pruritus?

▪ Have penicillins been taken and tolerated since then? If yes, which agents (if known)?

Table S1: Risk stratification according to local guideline for penicillin allergy management

Risk	Characteristics	Action
**Intolerance/ Low-risk**	• Isolated GIT-Symptoms<br>• Headache<br>• Unknown reaction in childhood<br>• Sleepiness<br>• Positive family history<br>• Chills	• Direct Delabeling: Removal of allergy<br>• Update medical records<br>• Inform patients (and family members)<br>• Inform general practitioner<br>• Include note in discharge letter
**Moderate risk**	• Rash without blistering<br>• Isolated dyspnoea<br>• Dizziness<br>• Angioedema<br>• Arrhythmia<br>• Isolated Hypotension<br>• Unspecified reaction occurring more than 10 years ago	Acute setting:<br>• No penicillins<br>• Therapy with alternative non-penicillin antibiotics<br>Non-acute settings:<br>• Evaluation by infectious disease specialists and/or pharmacists<br>• Interdisciplinary decision regarding allergological evaluation or oral provocation test
**High risk**	• Organ failure<br>• Anaphylaxis III + IV<br>• Serum sickness<br>• Blistering rash<br>• Stevens-Johnson syndrome (SJS)<br>• DRESS syndrome<br>• Erythema multiforme	• No penicillins<br>• Therapy with alternative non-penicillin antibiotics
Abbreviations: GIT: Gastrointestinal tract; DRESS: Drug rash with eosinophilia and systemic symptoms		

Supplementary 2: Protocol for direct oral challenge (DOC)

1.Eligibility criteria:

Inclusion criteria:

- Low to moderate risk of penicillin allergy
    - Risk stratification performed (pharmacy)
    - Verification of risk stratification by medical history (infectious diseases)

Exclusion criteria:

- Intake of antihistamines*
- Intake of corticosteroids at a dose >10 mg prednisolone equivalent
- Inability to provide informed consent
- Age <18 years

*If applicable, antihistamines may be discontinued for 72 hours and the direct oral provocation (DOP) performed after 72 hours.

2. Performance of the Direct Oral Provocation (DOP)

Infectious Diseases / Ward Physicians:

- Verification of medical history and eligibility criteria; patient information and consent (oral and/or written)
- Informing the ward team (physicians/nursing staff)
- Documentation of the procedure and course in the infectious diseases progress notes
- Instruction to the ward physician to prescribe amoxicillin (may also be done via email)

Nursing staff:

- Administration of amoxicillin 250 mg or 500 mg orally
- Measurement and documentation of vital signs (oxygen saturation, blood pressure, heart rate) at 30 and 60 minutes

Pharmacy:

If no reaction occurs:

- Delabeling with appropriate documentation (discharge letter and Excel database)
- Patient counseling regarding the implications of delabeling
- Important: patients should be informed that a delayed skin exanthema may still occur with prolonged exposure (<5%), but this does not represent a dangerous reaction
- If antibiotic therapy is indicated: switch to a beta-lactam antibiotic (if necessary, in consultation with infectious diseases)

If a reaction occurs:

- Assessment of the reaction (Type I vs. Type IV hypersensitivity)
- Treatment by the ward team
- Documentation of the reaction

3. Ensuring Delabeling Documentation

- Documentation of the oral challenge in the clinical progress notes (by infectious diseases)
- Documentation in the electronic patient record or medication chart
- Documentation in the discharge letter (suggested paragraphs were provided in the SOP)

Supplementary 3: Paper-based risk assessment documentation used by pharmacists

☐ Low
☐ Moderate
☐ High

PEN-FAST Score

☐ Very low risk
☐ Low risk
☐ Moderate risk
☐ High risk

Planned Intervention

☐ Direct delabeling
☐ Oral challenge

Patient Delabeled?

☐ Yes
☐ No → Reason:

Supplementary 4: Short Questionnaire to Assess Barriers and Facilitators in the implementation of penicillin allergy delabeling among healthcare professionals

1. Which professional group do you belong to?
☐ Physician
☐ Pharmacist
☐ Student
☐ Nurse / Healthcare professional
☐ Other: ____________________

2. Department / Specialty:

3. Do you consider penicillin allergy delabeling to be important and beneficial?
☐ Yes
☐ No

4. How do you assess the time required for the delabeling process?
☐ High time burden
☐ Appropriate time burden
☐ Minimal time burden

5. Are there any factors within the delabeling process that interfere with your workflow or cause delays?
☐ Yes
☐ No
If yes, please specify which factors or process steps:

6. Which aspects of the implemented delabeling process do you perceive as limiting for your professional group or routine clinical practice?
What does not work well?

7. Which factors contribute to the successful and feasible implementation of the delabeling process on your ward?
What works well?

Supplementary 5: Follow-up questionnaire for primary care physicians

This questionnaire was used to assess the sustainability of penicillin allergy delabeling in primary care.

1. Are you aware of the patient's penicillin allergy?
   - ☐ Yes
   - ☐ No

2. Was the result of the penicillin allergy evaluation documented in the discharge letter from our hospital?
   - ☐ Yes
   - ☐ No
   - ☐ I did not receive a discharge letter

3. What is the patient's current allergy status in your records?
   - ☐ The patient has a penicillin allergy
   - ☐ The patient does not have a penicillin allergy

4. Have you prescribed penicillin antibiotics to the patient since his/her hospital stay?
   - ☐ Yes
     If yes, please specify: ________________________
   - ☐ No
     If no, please indicate a reason: _________________

   If yes, did the patient tolerate the antibiotic?

   - ☐ Yes
   - ☐ No, reaction: ____________________
   - ☐ Unknown

5. Would you prescribe a penicillin antibiotic to the patient in the future?
   - ☐ Yes
   - ☐ No

   If not, what are the reasons? _____________________

Supplementary 6: Outcomes of risk stratification and delabeling regarding PEN-FAST:

According to PEN-FAST, 65 patients (36.3 %) were categorized as very low risk (PEN-FAST score 0), 43 patients (24.0 %) as low risk (score 1–2), 25 patients (14.0 %) as moderate risk (score 3), and 16 patients (8.9 %) as high risk (score 4–5). In 30 patients (16.8 %), calculation of the PEN-FAST score was not applicable due to documented previous tolerance of penicillins or a history of amoxicillin–Epstein–Barr virus–associated exanthema. Among patients delabeled directly, without further testing, PEN-FAST scores were 0 (n = 12) or 1 (n = 2), or delabeling was based on documented previous tolerance to penicillin antibiotics. PEN-FAST scores among patients undergoing provocation testing were 0 (n = 13), 1 (n = 5) and 4 (n= 1). The patient with PEN-FAST score 4 reported hypotension, diarrhea and vomiting following amoxicillin intake two years ago but was admitted with ongoing therapy with piperacillin-tazobactam. Therefore the patient could be delabeled. In two patients, the score was not applicable due to previous tolerance (n = 1) or Epstein–Barr virus–associated exanthema (n = 1). Among non-delabeled low- and moderate risk patients, 27 of 108 (25.0 %) had a PEN-FAST score of 3-5.
