# Supplementary material for: Penicillin allergy delabeling in a German University Hospital—implementation in inpatient settings: the PenGuin Pilot Study
Source: Infection. 2026 Aug 11;54(5):2715–24. doi: 10.1007/s15010-026-02915-7 (PMC13727940; doi:10.1007/s15010-026-02915-7)
Supplement: Supplementary file 2 [file 15010_2026_2915_MOESM2_ESM.pdf]

SQUIRE-Checklist: **Pen**icillin Allergy Delabeling in a **G**erman **U**niversity Hospital – Implementation in **In**patient Settings: the **PenGuin** Pilot Study

**Revised Standards for Quality Improvement Reporting Excellence (SQUIRE 2.0)**		
**Text Section and Item Name**	**Section or Item Description**	**Page, Line**
**Title and Abstract**		
1. Title	Indicate that the manuscript concerns an initiative to improve healthcare (broadly defined to include the quality, safety, effectiveness, patient-centeredness, timeliness, cost, efficiency, and equity of healthcare)	Page 1, Line 1-2
2. Abstract	a. Provide adequate information to aid in searching and indexing<br>b. Summarize all key information from various sections of the text using the abstract format of the intended publication or a structured summary such as: background, local problem, methods, interventions, results, conclusions	Page 2, Line 45-70
**Introduction**	Why did you start?	
3. Problem Description	Nature and significance of the local problem	Page 3, Line 72-77
4. Available knowledge	Summary of what is currently known about the problem, including relevant previous studies	Page 3, Line 77-94
5. Rationale	Informal or formal frameworks, models, concepts, and/or theories used to explain the problem, any reasons or assumptions that were used to develop the intervention(s), and reasons why the intervention(s) was expected to work	Page 3, Line 77-94
6. Specific aims	Purpose of the project and of this report	Page 3-4, Line 94-97
**Methods**	What did you do?	
7. Context	Contextual elements considered important at the outset of introducing the intervention(s)	Page 4, Line 97-117
8. Intervention(s)	a. Description of the intervention(s) in sufficient detail that others could reproduce it<br>b. Specifics of the team involved in the work	Page 4-5, Line 119-142
9. Study of the Intervention(s)	a. Approach chosen for assessing the impact of the intervention(s)	Page 5, Line 144-162

	b. Approach used to establish whether the observed outcomes were due to the intervention(s)	
10. Measures	a. Measures chosen for studying processes and outcomes of the intervention(s), including rationale for choosing them, their operational definitions, and their validity and reliability<br>b. Description of the approach to the ongoing assessment of contextual elements that contributed to the success, failure, efficiency, and cost<br>c. Methods employed for assessing completeness and accuracy of data	Page 5, Line 144-162
11. Analysis	a. Qualitative and quantitative methods used to draw inferences from the data<br>b. Methods for understanding variation within the data, including the effects of time as a variable	Page 6-7, Line 164-175
12. Ethical Considerations	Ethical aspects of implementing and studying the intervention(s) and how they were addressed, including, but not limited to, formal ethics review and potential conflict(s) of interest	Page 7, Line 177-179
**Results**	What did you find?	
13. Results	a. Initial steps of the intervention(s) and their evolution over time (e.g., time-line diagram, flow chart, or table), including modifications made to the intervention during the project<br>b. Details of the process measures and outcome<br>c. Contextual elements that interacted with the intervention(s)<br>d. Observed associations between outcomes, interventions, and relevant contextual elements<br>e. Unintended consequences such as unexpected benefits, problems, failures, or costs associated with the intervention(s).<br>f. Details about missing data	Page 7-16, Line 181-280
**Discussion**	What does it mean?	
14. Summary	a. Key findings, including relevance to the rationale and specific aims<br>b. Particular strengths of the project	Page 17, Line 283-290
15. Interpretation	a. Nature of the association between the intervention(s) and the outcomes	Page 17-20, Line 291-367

	b. Comparison of results with findings from other publications<br>c. Impact of the project on people and systems<br>d. Reasons for any differences between observed and anticipated outcomes, including the influence of context<br>e. Costs and strategic trade-offs, including opportunity costs	
16. Limitations	a. Limits to the generalizability of the work<br>b. Factors that might have limited internal validity such as confounding, bias, or imprecision in the design, methods, measurement, or analysis<br>c. Efforts made to minimize and adjust for limitations	Page 20-21, Line 369-392
17. Conclusions	a. Usefulness of the work<br>b. Sustainability<br>c. Potential for spread to other contexts<br>d. Implications for practice and for further study in the field<br>e. Suggested next steps	Page 21, Line 394-405
**Other information**		
18. Funding	Sources of funding that supported this work. Role, if any, of the funding organization in the design, implementation, interpretation, and reporting	Page 22, Line 418-419
